# Post‐translational modifications of proteins in cardiovascular diseases examined by proteomic approaches

**DOI:** 10.1111/febs.17108

**Published:** 2024-03-05

**Authors:** Miroslava Stastna

**Affiliations:** ^1^ Institute of Analytical Chemistry of the Czech Academy of Sciences Brno Czech Republic

**Keywords:** cardiovascular disease, MS‐based proteomics, post‐translational modifications, proteins

## Abstract

Over 400 different types of post‐translational modifications (PTMs) have been reported and over 200 various types of PTMs have been discovered using mass spectrometry (MS)‐based proteomics. MS‐based proteomics has proven to be a powerful method capable of global PTM mapping with the identification of modified proteins/peptides, the localization of PTM sites and PTM quantitation. PTMs play regulatory roles in protein functions, activities and interactions in various heart related diseases, such as ischemia/reperfusion injury, cardiomyopathy and heart failure. The recognition of PTMs that are specific to cardiovascular pathology and the clarification of the mechanisms underlying these PTMs at molecular levels are crucial for discovery of novel biomarkers and application in a clinical setting. With sensitive MS instrumentation and novel biostatistical methods for precise processing of the data, low‐abundance PTMs can be successfully detected and the beneficial or unfavorable effects of specific PTMs on cardiac function can be determined. Moreover, computational proteomic strategies that can predict PTM sites based on MS data have gained an increasing interest and can contribute to characterization of PTM profiles in cardiovascular disorders. More recently, machine learning‐ and deep learning‐based methods have been employed to predict the locations of PTMs and explore PTM crosstalk. In this review article, the types of PTMs are briefly overviewed, approaches for PTM identification/quantitation in MS‐based proteomics are discussed and recently published proteomic studies on PTMs associated with cardiovascular diseases are included.

## Graphical abstract

Mass spectrometry (MS)‐based proteomics has proven to be a powerful method capable of mapping, localizing and quantifying post‐translational modifications (PTMs). PTMs play regulatory roles in protein functions/activities and the recognition of PTMs specific to cardiovascular pathologies is crucial for usage in clinics. With sensitive MS instrumentation, novel computational strategies, machine learning and deep learning methods, PTMs beneficial or unfavorable effects on cardiac function can be determined.

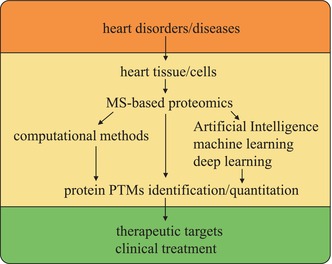

**Abbreviations**
AAD: aortic aneurysm and dissection
AF: atrial fibrillation
AI: artificial intelligence
Cal‐1: calsarcin‐1
cTnI: cardiac troponin I
cTnT: cardiac troponin T
DHHC: Asp‐His‐His‐Cys domain‐containing
ENH2: enigma homolog 2
FT: Fourier transform
FT‐ICR: Fourier transform ion cyclotron resonance
GA: glycated‐human serum albumin
GS M4: glutathione synthetase mutant
HF: heart failure
HFpEF: heart failure with preserved ejection fraction
I/R: ischemia/reperfusion injury
ICM: ischemic cardiomyopathy
I‐HF/NI‐HF: ischemic heart failure/non‐ischemic heart failure
KO: knockout
MLP: LIM protein
MPG: *N*‐2 mercaptopropionylglycine
MS: mass spectrometry
MS/MS: tandem mass spectrometry
MRM/SRM: multiple reaction monitoring/selective reaction monitoring
NF: non‐failing
PLMD: protein lysine modification database
PTM: post‐translational modification
ROS: reactive oxygen species
SERCA2a: sarcoplasmic/endoplasmic reticulum calcium ATPase 2a
SIRT1: silent information regulator 1
SR: sinus rhythm
TAA: thoracic aortic aneurysm
TAC: transverse aortic constriction
TAD: thoracic aortic dissection
TMT: tandem mass tag
β‐MHC: β‐myosin heavy chain

## Introduction

The proteomes comprise proteins that are post‐translationally modified and these proteins can contain single or multiple post‐translational modifications (PTMs) of the same type or various types. Different combination of multiple PTMs in the protein results in a large amount of proteoforms (protein forms of single gene) [1, 2, 3] that contribute to protein functional diversity [4]. PTMs have an important role in the regulation of activity, function, localization and conformation of proteins and they dynamically control the proteins under both physiological and pathological conditions. In addition, PTMs can influence interactions with other molecules and trigger specific protein–protein interactions [5]. Various PTMs can work together within a single protein or among multiple proteins in PTM crosstalk [6, 7, 8].

Clarification of the mechanisms underlying various PTMs at molecular levels is crucial for understanding of the pathological outcomes. Recognition of the specific PTM type and localization within protein amino acid sequence can determine functional significance [9]. The characterization of disease‐specific PTMs can lead to identification of novel strategies for disease prognosis, diagnosis and treatment. Multiple reviews have been published recently that summarized PTMs roles in the pathogenesis of various diseases, such as acetylation, carbamylation and citrullination in rheumatoid arthritis [10], lysine acetylation in human pathologies [11] and neurodegenerative disease [12], pathological consequences of arginine citrullination in the formation of inflammatory diseases [13], ubiquitination in cancer and neurodegenerative disorders [4], sumoylation after brain injury [14] and in neurodegenerative diseases [15], succinylation in cancer [16], multiple PTMs in colorectal cancer [17], O‐glycosylation in gastrointestinal tumors [18], and acetylation, arginylation, lipidation and ubiquitination of protein N‐ and C‐termini [19]. In addition, review articles that targeted PTMs of specific proteins have been published, with respect to PTMs of thyroglobulin in different thyroid pathologies [20], methylation of Tau protein [21] and PTMs of the p53 protein in Alzheimer's disease [22], phosphorylation, glycosylation, palmitoylation and ubiquitination of G protein‐coupled receptors superfamily [23], and effects of PTMs of α‐synuclein aggregation in neurodegenerative disorders [24]. Furthermore, uncommon PTMs have been discussed, such as AMPylation of small GTPases by Fic enzymes [25], ADP‐ribosylation [26, 27], tyrosine nitration and tyrosine sulfation [27], and crotonylation role in DNA damage response [28].

Post‐translational modifications associated with cardiovascular diseases encompass acute, chronic and genetic etiologies [ischemic heart disease, hypertension, heart failure (HF), various cardiomyopathies, etc.] [29]. In recent years, the number of papers with PTMs related to cardiovascular diseases has increased. In addition to phosphorylation, glycosylation and acetylation (the most studied PTMs in the heart), studies related to other PTMs and their involvement in cardiovascular disease modulation have been reported, such as malonylation, butyrylation, propionylation, crotonylation, glutarylation and succinylation [30]. For example, malonylation has been shown to have regulatory function in various diseases (metabolic disorders, immune regulation, inflammation, etc.) [31] and, importantly, malonylation‐associated enzymes have been demonstrated to have close relationships to cardiovascular pathologies [30, 32]. In addition, the growing importance of glutathionylation (S‐glutathionylation) in cardiac function has been recognized and several strategies have been developed for proteomic identification of glutathionylated proteins [33]. Citrullination is another PTM that has been studied in heart pathologies [34, 35]. Nevertheless, protein site‐specific identification and quantitation of PTMs in cardiac cells and tissues, as well as identification of molecular targets for heart disease treatments, has been rather limited despite advances in technologies developed in recent years.

Proteomic methods and specifically mass spectrometry (MS)‐based proteomic approaches have demonstrated great potential in the detection, localization and crosstalk of PTMs in many pathological events including cardiovascular diseases. Moreover, because classical biochemical methods may not be sufficient for global profiling of PTMs and characterization of protein PTM functionalities, they have shifted to development of computational proteomic strategies [36]. Computational methods that can predict PTM sites based on MS data have been developed [6, 9, 36] and, more recently, machine learning and deep learning (advanced machine learning) methods in which various available databases can be used to train a model for PTM prediction have gained increasing interest [37, 38, 39, 40, 41, 42]. For example, the UniProt database with PTM annotations [43], protein lysine modification database (PLMD) 3.0 [44], dbPTM database [45, 46], PhosphositePlus [47] and protein S‐palmitoylation database SwissPalm 2 [48] have been used. The major online PTM databases and related tools have been published and summarized [37, 44, 49], including the challenges of computational methods for PTM prediction.

In this review, the types of PTMs were briefly overviewed, approaches for PTM identification/quantitation in MS‐based proteomics are described and examples of proteomic studies on PTMs associated with cardiovascular diseases that were published from 2018 onward are discussed.

## Types of PTMs

PTMs appear on C‐ or N‐termini of proteins and/or at protein amino acid side chains and they can range from small chemical modifications to addition of entire proteins/molecules. Generally, PTMs are reversible (contain covalent modification) or irreversible (contain proteolytic modification). Furthermore, PTMs can be enzymatic (mediated by enzyme), chemical (triggered by chemical changes in local environment) and physical (protein cleavage and degradation) [29]. For example, protein glycosylation, phosphorylation and acetylation belong to enzymatic reversible PTMs, whereas deamidation is chemical irreversible PTM. In addition, reversible (signaling) and irreversible (oxidative damage) redox PTMs that can occur, for example, by reaction of protein amino acids with reactive oxygen species (ROS)/reactive nitrogen species during oxidative stress, are other forms of PTMs (reversible cysteine S‐nitrosylation, disulfides, S‐sulfenylation and irreversible cysteine‐sulfinic and cysteine‐sulfonic acid) [50]. Various PTMs contribute to regulation of protein function and, because a dynamic mixture of protein modified and unmodified forms may be present simultaneously, they increase the functional variability of the proteome as well. The updated dbPTM database from 2022 (https://awi.cuhk.edu.cn/dbPTM) comprises over 70 types of protein PTMs with over 2 235 600 PTM sites confirmed experimentally [46].

The 10 most studied PTMs are phosphorylation, acetylation, ubiquitylation, methylation, glysosylation, sumoylation, palmitoylation, myristoylation, prenylation and sulfation. Detailed descriptions about these PTMs and their roles in various biological processes have been provided previously [49]. Frequently modified amino acids in proteins include lysine (32 PTM types) [46], cysteine (about 30 PTM types) [46] and serine (about 20 PTM types) [46]. Lysine possesses a broad variety of PTMs formed in different modification processes, such as acylation (addition of a functional group, such as succinylation, malonylation, propionylation [51], glutarylation and crotonylation), sumoylation, ubiquitination, neddylation (covalent linkage of protein modifiers) and glycation (non‐enzymatic attachment of a sugar molecule) [44].

In the heart, well‐studied PTMs, such as phosphorylation, glycosylation and acetylation, have important roles in cardiac function. However, only a small number of other known types of PTMs have been studied in cardiac pathologies. For example, a relatively new PTM, crotonylation, has been recognized as a key regulator of proteins in cardiovascular disorders [52, 53]. Crotonylation belongs to PTMs in which a crotonyl group from crotonyl‐CoA is reversible and covalently added to a lysine residue of the protein. Crotonylation was identified as a new type of PTMs on histones [54] in 2011 and on non‐histone proteins in 2017 [55]. A new bioinformatic tool has been described to predict crotonylation sites on human non‐histone proteins [56]. Review articles have been published dealing with crotonylation in biomedicine [57], the challenges associated with crotonylation identification, regulation and biological implications [58], crotonylation function [59, 60] and related pathologies [60], and the protein lysine crotonylation perspective [61].

Another PTM that has gained an interest recently in studies of cardiovascular pathologies is citrullination (arginine deamination) [34, 35]. It is a type of PTM that is catalyzed by peptidyl arginine deiminase enzymes. These enzymes are activated by high calcium concentration and they irreversibly convert arginine in protein into citrulline. Citrullination decreases the overall charge of the protein, which leads to protein structural changes and, subsequently, it can alter protein binding features. Furthermore, acidity of the amino acid side chain is changed during citrullination as the basic pI of arginine (11.4) changes to the pI 5.9 of citrulline [62], which leads to alteration of protein function. More details can be found in reviews concentrating on the role of citrullination in targeting potential biomarkers in cardiovascular diseases [34] and identification of citrullination and citrullination sites by novel approaches in MS‐based proteomics [35].

Recently, the implications for sarcomere proteins (cardiac myosin binding protein C, actin, cardiac troponin and titin) as substrates for glutathionylation in cardiac pathologies have been published [63]. For example, the question of ‘whether serum levels of S‐glutathionylated‐cMyBP‐C (cardiac myosin binding protein C) could be employed as an important clinical tool in patient stratification, early diagnosis in at risk patients before HFpEF’ was discussed [63]. HFpEF stands for ‘heart failure with preserved ejection fraction’, a form of a clinical syndrome in which the left heart ventricle is stiffened and as a result, left ventricular relaxation is dysfunctional during diastole [64].

In addition, only a small number of cardiac proteins was known to be reversible S‐palmitoylated, with no information about the extent of protein S‐palmitoylation (lipid modification) in the heart. Palmitoyl chain (fatty acyl chain) is covalently attached to cysteine thiol group of target protein and S‐palmitoylation is mediated by Asp‐His‐His‐Cys domain‐containing (DHHC) enzymes [65]. A recent study [65] reported high‐throughput experiments using MS‐based proteomics for determination of the scope of protein S‐palmitoylation and its regulation by DHHC enzymes in the heart.

## Approaches for PTM identification/quantitation with focus on MS‐based proteomics

Over 400 different types of PTMs have been reported [66] and over 200 various types of PTMs have been discovered using MS‐based proteomics [45, 67]. MS‐based proteomics has proven to be a powerful method capable of global PTM mapping with the identification of modified protein/peptide, the localization of PTM site and PTM quantitation. In addition to routinely applied HPLC–tandem MS (MS/MS), alternatively, capillary electrophoresis‐MS/MS has been used in electrophoretic modes such as capillary zone electrophoresis or capillary isoelectric focusing [68, 69]. For MS‐based PTM analysis, bottom‐up (short proteolytic peptides are analyzed; proteolysis) [70], middle‐down (long proteolytic peptides are analyzed; limited proteolysis) [71, 72] and top‐down (intact proteins are analyzed; no proteolysis) [73] strategies have been applied. Information and data about PTMs have been constantly accumulated into various databases, either collected from experiments or predicted from manually curated high quality data.

### MS‐based proteomic workflows

The important and crucial step in targeted PTM analysis is the preparation of quality samples prior their introduction to MS instruments. To exploit the capabilities of high efficient and sensitive MS technology, the complex samples are usually pretreated to ensure efficient and reliable mapping of PTM proteomes. In the MS‐based bottom‐up (shotgun) proteomic workflow, proteins isolated from cell/tissue sample can be fractionated to reduce sample complexity (by SDS/PAGE, chromatographic techniques or precipitation), followed by digestion of separated proteins, or directly digested by various enzymes and their combination. Because many PTMs are present in low abundances, resulted proteolytic PTM peptides are usually enriched using various techniques (e.g. acylated peptides by antibodies, phosphopeptides by immobilized metal affinity chromatography/TiO_2_ and glycopeptides by lectin). Enriched PTM peptides are analyzed by HPLC–MS/MS (or capillary electrophoresis‐MS/MS) with a subsequent search of MS spectra against protein databases for identification of peptide sequences, precise PTM locations and quantification [70]. Generally, a data‐dependent acquisition strategy in HPLC–MS/MS analysis is used (i.e. a first survey scan is performed on a sample with proteolytic peptides followed by selection of a number of most intense peptide precursor ions for further MS/MS fragmentation). A review on traditional affinity‐based strategies for profiling of PTMs has been published [36] and enrichment strategies for PTM characterization in MS‐based proteomics analysis have been reviewed [70, 74]. For example, affinity strategies have been discussed [74], such as immunoenrichment, chromatography and protein pull‐down, as well as chemical enrichment strategies, including metabolic and chemoenzymatic labelling and capture by chemoselective probes. In addition, a tutorial for optimizing HPLC parameters in HPLC–MS/MS has been written for bottom‐up proteomics [75] and a review has been published dealing with HPLC–MS/MS detection for the bottom‐up analysis of complex proteomic samples [76].

MS‐based top‐down proteomics is a method for the analysis of whole protein molecules in which mass spectra of intact proteins are measured, the most abundant peaks are chosen for fragmentation and tandem mass spectra of the fragment ions are acquired [73, 77]. The method preserves PTM motifs in post‐translationally modified proteins that can be otherwise lost in bottom‐up proteomics because protein identification is obtained from fragmentation of the intact protein and digestion of protein into peptides is avoided [3, 77, 78]. Top‐down proteomics has advantage of accurate intact protein mass measurement, mapping of PTMs with full coverage and determination of the order of multiple PTMs in an amino acid sequence. Usually, size‐based fractionation of complex protein mixture is performed before MS analysis to separate large proteins (30–200 kDa) from the presence of low‐molecular mass proteins (5–20 kDa) and avoid signal suppression by co‐elution of abundant low‐molecular mass proteins. In addition, Fourier transform (FT)‐based MS instruments are often applied that possess ultrahigh resolving power and high‐accuracy mass measurements [78, 79]. For example, MS‐compatible serial size exclusion chromatography has been used in direct combination with 12T FT‐ion cyclotron resonance (FT‐ICR) MS for targeted top‐down analysis of the protein extracts from human and swine cardiac tissue [78]. Herein, intact mass measurements of 31 resolved proteoforms containing PTMs (30–50 kDa) were obtained simultaneously from a single spectrum with subsequent amino acid sequence characterization. Furthermore, software has been developed to enhance the identification of large modified proteins in complex systems [77].

The middle‐down approach integrates some advantages of both top‐down and bottom‐up techniques. It can yield the peptides that contain multiple PTMs and thus provides more detailed data about co‐existing PTMs than the bottom‐up method and without technical challenge presented by the top‐down concept [71, 72, 73].

For proteins/peptides/PTMs quantitation, LC–MS/MS strategies include label‐free techniques [80, 81], isotope labeling techniques via metabolic approaches (stable isotope labeling by amino acids in cell culture) [82], isobaric labeling approaches (isobaric tags for relative and absolute quantification) [83], tandem mass tags (TMT) [84] and targeted quantitation [multiple reaction monitoring/selective reaction monitoring (MRM/SRM)] [85]. Various quantitative proteomic approaches for redox PTMs have been reviewed [86]. MRM/SRM is a sensitive assay used in targeted MS‐based proteomics for both antibody‐free validation of PTMs and quantification of PTMs. It complements the discovery features of shotgun MS by its potential for reliable quantitation of low abundant analytes in complex mixtures [85]. In the MRM/SRM assay, selected peptides that represent a protein in a complex sample are quantified. First, predefined *m*/*z* values are specifically selected that correspond to the precursor peptide ion and a specific fragment ion of the peptide. Then, a series of specific pairs of precursor ions and fragment ions (transitions) are monitored over time for precise quantitation and, in combination with the retention time of the targeted peptide and signal intensity of the specific transition, they create the assay [85]. The method was used for the characterization and quantification of site‐specific protein phosphorylation in murine and human heart mitochondria [87] and was reviewed recently for PTM quantitation in cardiology [88].

4D quantitative proteomics is a novel technology in bottom‐up LC–MS/MS that incorporates the fourth separation dimension, ion mobility, in addition to traditional retention time, mass‐to‐charge ratio and ion intensity dimensions. It allows detection of low abundant proteins in complex samples with high sensitivity, which are otherwise difficult to identify by the 3D proteomic approach [89, 90]. Recently, the software that extracted the data obtained from these four dimensions has been introduced and is available (http://maxquant.org) [89]. The method has been used for clarification of the effect of succinylation in aortic aneurysm and dissection [91].

### Advances in PTM identification and PTM prediction

In shotgun proteomics, the peptide identification is usually based on the search of acquired MS/MS spectra against protein sequence databases by use of peptide identification algorithms [92, 93]. The various databases of PTMs are available; for example, UniProt database with PTM annotations (UniProt Consortium) [43], PLMD 3.0 [44] (an expanded database that also includes previous data from compendium of protein lysine acetylation CPLA 1.0 [94] and updated compendium of protein lysine modifications CPLM 2.0 [95]; http://plmd.biocuckoo.org), dbPTM database [45, 46] (https://awi.cuhk.edu.cn/dbPTM), and PhosphositePlus [47] (http://www.phosphosite.org). SwissPalm 2 (https://swisspalm.org) [48, 96] is a database on protein S‐palmitoylation with 38 palmitoyl‐proteomes from 17 different species.

Traditional ‘closed’ database searches used in LC–MS/MS analysis require prior knowledge about peptide PTMs present in examined samples, and user‐predefined subsets of PTMs have to be included in the initial search parameters. In addition, a narrow precursor mass tolerance is used for a search. However, using this method, large parts of spectra were not assigned, with many of them representing the peptides with PTMs [97, 98, 99] and thus many PTMs remained undetected. Novel methods have been developed to enhance identification of diverse PTMs and speed identification process. One of them is an ‘open’ search strategy. Compared to conventional ‘closed’ database searches, in an ‘open’ search strategy (mass‐tolerant search), wider precursor mass tolerances are applied in database searches (hundreds of daltons) and both known and unknown PTMs can be identified. However, a postprocessing tool was required for the identification of PTM type and its localization within a peptide sequence. ‘Open’ database searches detected the peptides with any mass shift that may correspond to a single modification or a combination of several modifications [100]. Various ‘open’ searches tools have been reported; for example, MSFragger, an algorithm for identification of peptides and their modified forms [93]. It uses a novel fragment‐ion indexing method with ‘open’ database searches. Using of MSFragger on large proteomic datasets (containing millions of MS/MS spectra), ultrafast peptide identification (over 100‐fold increase in speed) and enhancement of modification rates across different experimental samples was reported [93] in comparison with existing proteome database search tools. MSFragger was applicable to quantitative proteomics with labeling‐based experiments and its use in affinity purification experiments with an approximately 300% increase in the identification of MS spectra per enriched protein was demonstrated [93]. Another automated bioinformatics tool presented recently was PTM‐Shepperd [100] that characterized PTMs from ‘open’ search peptide spectra, based on fragmentation spectra similarity, amino acid localization, relative modification rates and shifts in retention times. It was suitable for comparative analysis in multi experiment settings where PTM profile alteration were evaluated under varied experimental conditions or between different laboratories. Comet‐PTM was the next ‘open’ search algorithm that was used for identification of modified forms of peptides by PTMs [101]. Comet‐PTM efficiently resolved the mass shift produced by the modification in the fragmentation series of MS spectra and assigned PTM to correct amino acid residue, thus doubling the number of modified peptides identified by a conventional ‘open’ search strategy [101]. The identification performance of two ‘open’ search methods, MSFragger and Comet‐PTM, has been compared recently to a new ‘open’ search Comet‐ReCom algorithm (an improved Comet‐PTM) [102]. The Comet‐ReCom method increased peptide identification performance of Comet‐PTM by 68% as a result of its capability to minimize the effects of experimental precursor mass errors, as well as use a semi‐supervised approach for an ultra‐tolerant mass window database search [102].

Recently, PTMselect has been designed and its capabilities in PTM discovery by MS‐based proteomics were demonstrated using phosphorylation as the example of PTM [103]. PTMselect was described as a digestion‐simulating software that computationally selects and suggests the optimal set of proteases for optimal coverage of targeted or global PTMs on one protein or multiple proteins [103]. As the example in [103], six phosphoproteins (p53, huntingtin, programmed cell death 1 protein, citron‐kinase, lamin and cortactin; mass range from 31.6 to 347.6 kDa) and eight proteases (trypsin/P, Lys‐C, Lys‐N, chymotrypsin, Arg‐C, Asp‐N, V8‐E and V8‐DE) and CNBr were chosen, and the number of phosphosites identifiable by MS experiment was calculated by PTMselect to evaluate the effects of various enzymes. Using PTMselect simulation on these six proteins in parallel digestion setting showed that phosphosite coverage can be improved by up to 35% and 37% when using parallel digestion with two proteases and three proteases, respectively (as compared to digestion with only trypsin). Common and advanced data analysis strategies for protein modification identification in MS‐based proteomics can be found in review [104].

Artificial intelligence (AI) refers to the intelligence of machines/computers that are able to simulate human intelligence. The technologies such as machine learning and deep learning are the subsets of AI, focusing on development of computer systems that can automatically learn and improve performance based on MS experiments or data. With increasing expansion in size and complexity of proteomic data, the concept of integrated machine‐learned model of a proteomic experiment has been suggested and discussed. Ultimately, the model should integrate all experimental steps in proteomic workflow, from the sampling of the biological system to tandem MS/MS [38]. Up to now, machine learning as well as deep learning [42] were applied to individual steps of proteomic experiments; for example, chromatography analysis (DeepLC; a deep learning peptide retention time predictor for peptides with yet unseen modifications) [39], protein enzymatic digestion (DeepDigest; a sequence‐based deep learning algorithm for prediction of cleavage probability of each potential cleavage site on the protein sequences by 8 proteases) [40] and MS/MS analysis (Prosit; a deep neural network trained for prediction of peptide tandem mass spectra by deep learning) [41].

Recently, the AI‐based platforms and models have been designed and trained for prediction of protein PTMs in human diseases. For example, deep‐learning prediction model mind‐s [105] was able to predict 26 types of protein PTMs and their amino acid sites from two MS datasets consisting of around 50 000 proteins using protein sequences and structures. Because machine learning models utilized large‐scale datasets from public data repositories for training, the major challenge was experimental variability among these datasets. Thus, another study investigated the reusability of LC–MS datasets from standard bottom‐up proteomic experiments for machine learning applications [106]. The effect of variability of setup parameters in LC–MS experiments (data acquisition methods, biological systems and experimental designs) on machine learning performance was highlighted.

The contribution of AI to proteomics today and in the future has been summarized recently [107] and several applications can be mentioned here. For example, deep learning and machine learning can improve data analysis and data interpretation in terms of recognition of patterns in MS‐datasets, denoising complex spectra for better accuracy of identified proteins/peptides and detection of association of identified proteins with known pathologies. One of the main AI contributions can be seen in combination of proteomics data with the results obtained from other ‐omics methods, which are otherwise impossible to handle and identify by humans. Thus, a broad picture can be created about regulation, modification and interaction of biological systems. Furthermore, AI can help in early diagnosis and during disease progression by efficient and fast analysis of data. Furthermore, AI can identify the patients who are most susceptible to a specific therapy in personalized medicine [107].

More details about machine learning, deep learning and their application for prediction of PTM sites (acetylation, phosphorylation, ubiquitination, etc.) and PTM crosstalk within and across the proteins has been provided in recent review articles [6, 37, 38, 42, 108]. In addition, articles have been published that discuss the role of AI in proteomics and biomarker discovery [109], as well as AI tools for predicting PTMs [110].

## PTMs relation to heart and cardiovascular diseases

Post‐translational modifications can modulate pathological processes via regulation of protein activities, localizations, structures and stabilities [9]. Considering cardiovascular diseases, either chronic or acute, PTMs played important roles in ischemia/reperfusion (I/R) injury, hypertrophic and dilated cardiomyopathies, hypertension, fibrillation dysfunction, and HF. The review articles have been published that covered protein PTMs related to cardiovascular pathologies, such as ubiquitination [4, 111] and sumoylation [112, 113].

**Fig. 1:**
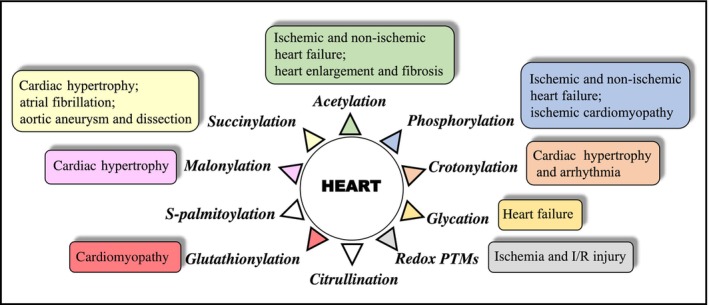
Schematically shown post‐translational modifications (PTMs) that are covered in PTMs relation to heart and cardiovascular diseases and their association with heart and cardiovascular diseases as identified by mass spectrometry (MS)‐based proteomics. Additional details can be found in the text.

The following sections map progress in the types of PTMs that have been studied from 2018 onward and are related to heart and cardiovascular disorders/diseases using MS‐based proteomic approaches either at the global proteome level and/or at individual protein levels (Fig. 1). The studies cover crotonylation, succinylation, malonylation, glutathionylation, redox SO_2_H/SO_3_H, S‐palmitoylation, phosphorylation, acetylation, citrullination and glycation.

### Global proteomic profiling

The role of relatively new type of protein PTM, lysine crotonylation [54], has been investigated in cardiac dysfunction and arrhythmia [52]. The effect of decrotonylation by silent information regulator 1 (NAD‐dependent protein deacetylase sirtuin‐1) (SIRT1) on heart rhythm and cardiac function was explored in a model of SIRT1 knockout (KO) mice. Sirtuin family proteins are the enzymes which are able to remove the modifications on lysine residues of target proteins, including crotonylation, through the process of NAD^+^‐dependent deacylation (deacetylation, demalonylation, desuccinylation, deglutarylation, etc.) and, in this way, they modulate and control biological functions and activities of target proteins [114, 115]. Using quantitative MS‐based proteomics and cell biology experiments [52], it was found that protein sarcoplasmic/endoplasmic reticulum calcium ATPase 2a (SERCA2a) in the heart tissue of SIRT1 KO mice was significantly crotonylated on Lys 120 compared to control tissue, which resulted in SERCA2a decreasing activity. As well, knockout of SIRT1 in mice led eventually to impaired cardiac function, cardiac hypertrophy and arrhythmia, implying the correlation between increased SERCA2a crotonylation mediated by SIRT1 and these pathological outcomes [52].

Other studies have targeted protein succinylation [91, 116, 117, 118]. In the study by Hershberger *et al*. [116], cardiac succinylome was characterized using MS‐based proteomics. The task was to determine whether previously observed accelerated development of cardiac dysfunction and increased mortality in a whole‐body SIRT5 KO mice model with pressure overload‐induced cardiac hypertrophy by transverse aortic constriction (TAC) surgery [119] was a result of a heart‐intrinsic (cardiomyocyte‐specific) effect of SIRT5 or a heart‐extrinsic SIRT5 effect (whole‐body). It has been documented previously that SIRT5 fulfilled a cardioprotective role and played essential part in maintaining cardiac function and survival by desuccinylation of the enzymes involved in cellular oxidative metabolism [119, 120]. In addition, it was suggested that regulation role of sirtuin was different between tissues, such that different degrees of malonylation were detected in SIRT5 KO between tissues [121]. In addition, diverse metabolic phenotypes have been observed between the whole‐body SIRT3 KO model [122] and the skeletal muscle‐specific SIRT3 KO model [123] with similar hyperacetylation profiles. Despite these observations, understanding of the contribution of individual tissues to the phenotypes remains to be determined. Thus, in the study by Hershberger *et al*. [116], the cardiomyocyte‐specific SIRT5 KO mouse model was created in which postnatal SIRT5 depletion resulted in gradually increased succinylation of the proteins for up to 30 weeks and, specifically, the proteins associated with oxidative metabolism between 15 and 31 weeks after SIRT5 ablation. Surprisingly, in contrast to increased mortality (i.e. decreased survival) in whole‐body SIRT5 KO mice compared to wild‐type controls with TAC, there was no difference in survival in heart‐specific SIRT5 KO mice with TAC surgery compared to their littermate controls. Thus, the phenotype of increased mortality was not repeated in a heart‐specific SIRT5 KO mouse model. It was implied that varied survival of SIRT5 KO mice may depend on a multi‐tissue effect of SIRT5 and/or prenatal effect of SIRT5 [116].

Succinylation has been hypothesized to regulate atrial energy metabolism during atrial fibrillation (AF) [117] (i.e. a heart condition with an irregular and often abnormally rapid heart rhythm). Previously, quantitative MS‐based proteomics has been used to profile and detect the alteration in the expression levels of energy metabolism‐associated proteins in atrial tissue (left atrial appendage) from patients undergoing valvular heart disease surgery and having sinus rhythm (SR) (without a history of AF; *n* = 6) or permanent AF (*n* = 8) [124]. In a study by Tu *et al*. [124], the majority of the proteins that were related to energy metabolism and expressed differently between SR and AF patients were down‐regulated. In extended work from the same lab group [118], quantitative MS‐based proteomics was utilized to identify the expression changes in succinylated proteins in the similar types of AF and SR tissue samples as in Tu *et al*. [124]. In the study by Bai *et al*. [118], the proteins that differed in expression levels between AF and SR (AF/SR fold changes ≥ 1.3) possessed 246 and 45 sites with up‐regulated and down‐regulated succinylation, respectively. Subjected to protein–protein interaction network, the succinylated proteins in AF were highly enriched in energy metabolism. The alteration in energy metabolism‐related protein levels and succinylation levels between AF and SR patients implied the association of these proteins with AF in valvular cardiac disease. Improving myocardial energy metabolism can be a way to treat AF [118].

In another study, proteomics was used to clarify the effect of succinylation in aortic aneurysm and dissection (AAD) [91]. Herein, the global profiles of proteome and succinylome in aorta samples of heart transplant donors (control; *n* = 5), patients with TAA (thoracic aortic aneurysm) (*n* = 5) and patients with TAD (thoracic aortic dissection) (*n* = 5) were evaluated using 4D label‐free MS‐based quantitative proteomics. Differentially succinylated proteins and succinylated sites between AAD and normal controls were compared. It was concluded that succinylated protein sites may be used as common targets in aortic aneurysm and aortic dissections as 120 differentially succinylated sites from 76 succinylated proteins overlapped between TAD and TAA groups (fold change > 1.5). In addition, an *in vitro* model of succinylation with vascular smooth muscle cells demonstrated that succinylation can modify enzymatic activity of metabolic enzymes because lactic dehydrogenase A and pyruvate kinase activities increased and succinate dehydrogenase A activity decreased after treatment of vascular smooth muscle cells with 50–100 mm sodium succinate. In conclusion, succinylation contributed to the aortic diseases and the proteins containing succinylated sites could function as potential markers and therapeutic targets [91].

MS‐based proteomics has been used for profiling of lysine malonylation in mice heart after TAC surgery‐induced cardiac hypertrophy [32]. It was identified that overall malonylation level was reduced in hypertrophic TAC hearts *in vivo* (measured 8 weeks after TAC) compared to sham. In addition, hypertrophic H9C2 cardiomyocytes *in vitro* (hypertrophy induced by treatment with angiotensin II for 48 h) exhibited decreased levels of malonylation compared to control cells as well. MS‐based proteomics quantified 172 malonylated sites and 150 malonylpeptides from 87 proteins in TAC mice. Approximately 46% of identified proteins possessed two or more malonylated sites; however, the protein with up to 30 malonylated sites was identified as well. Myocardial isocitrate dehydrogenase 2 exhibited a reduced malonylation in both hypertrophic hearts and hypertrophic cardiomyocytes compared to controls; however, the isocitrate dehydrogenase 2 expression level remained unchanged by cardiac hypertrophy both *in vivo* and *in vitro*. Such work reported for the first time the profile of protein malonylation as connected to cardiac hypertrophy, as well as association of cardiac hypertrophy with downregulation of protein malonylation.

**Fig. 2:**
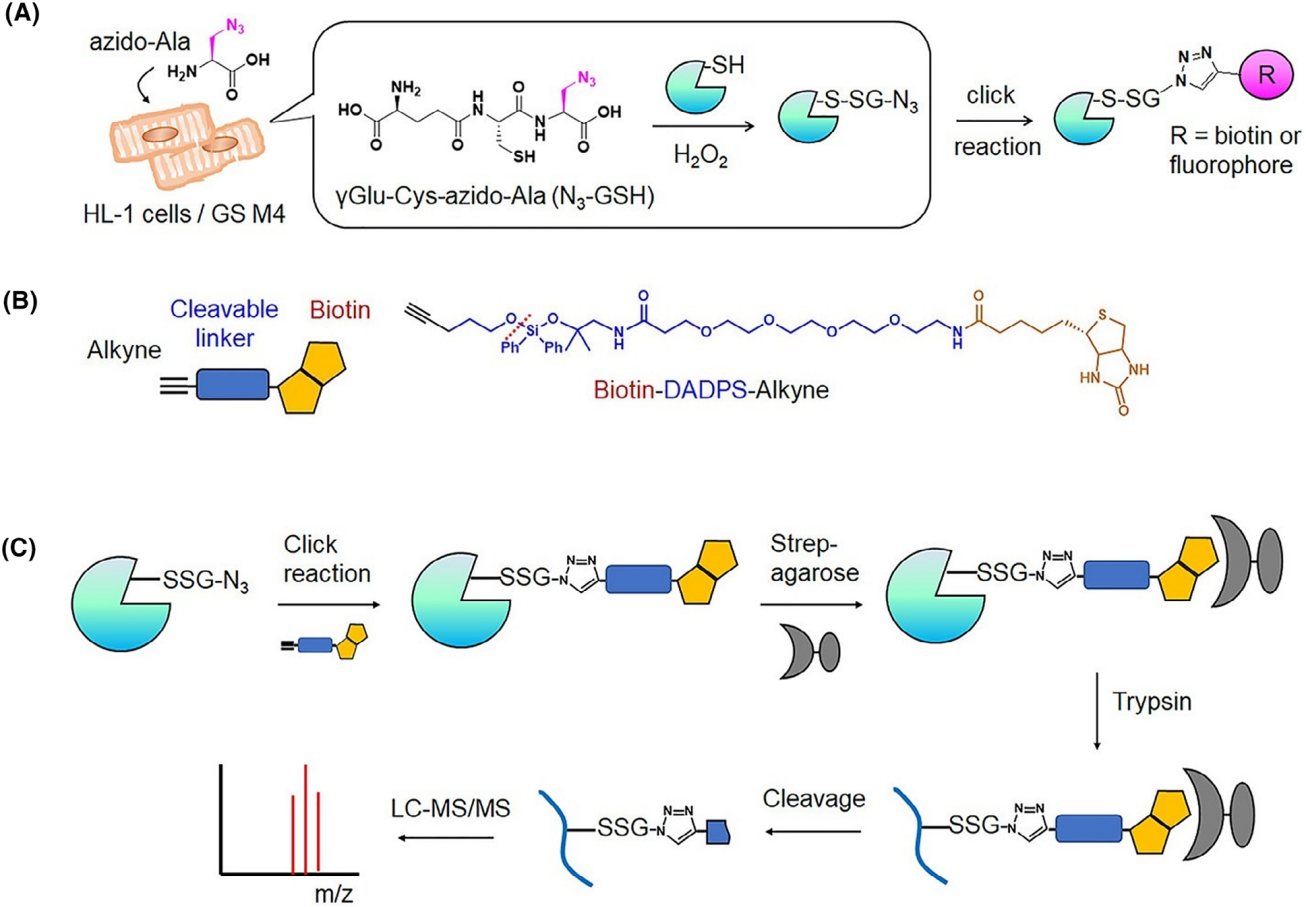
Schema of a clickable glutathione approach for identification of glutathionylated peptides/proteins by LC–MS/MS. (A) HL‐1 cells were transfected with a mutant of glutathione synthetase GSM4, which uses azido‐alanine to synthesize azido‐glutathione N_3_‐GSH. After addition of reactive oxygen species (ROS) stimulus (H_2_O_2_), glutathionylated proteins containing azide groups were enriched using a click reaction with the alkyne functional group of biotin‐alkyne. (B) formula of biotin‐DADPS‐alkyne used in the present study for isolation and elution of glutathionylated peptides. (C) After the click reaction, biotinylated glutathionylated proteins were bound into streptavidin‐agarose beads, then on‐bead digested by trypsin/Lys‐C, resulting in glutathionylated peptides eluted by acidic cleavage of DADPS linker and identified by LC–MS/MS. Additional details are provided in the text. Figure adapted from VanHecke *et al*. [33]; copyright (2019) American Chemical Society.

Recently, proteomic identification of protein glutathionylation in cardiomyocytes has been carried out [33]. Glutathionylation is a reversible PTM that causes protein oxidation on redox‐sensitive cysteine residues. It involves the formation of a disulfide bond between glutathione and cysteine residue of target protein as a response to various redox stimuli, such as enhanced oxidative stress and an elevated level of ROS in cells [33, 125]. In the study by VanHecke *et al*. [33], HL‐1 cells, which have a gene expression profile similar to adult atrial myocytes, were treated with glucose oxidase for 10 min (ROS stimulus; production of H_2_O_2_ by consuming glucose; corresponds to exposure of 0.5 mm of H_2_O_2_ per minute) to induce glutathionylation and the click chemistry method (cycloaddition of alkyne group to azide functional group) as reported previously [126, 127, 128] was used for identification of glutathionylated proteins. HL‐1 cells were first infected by adenovirus Ad‐GS M4 to express *in situ* high levels of glutathione synthetase mutant (GS M4). This was followed by incubation of the cells with azido‐alanine (0.6 mm, 24 h) to synthesize clickable azido‐glutathione (γGlu‐Cys‐azido‐Ala; N_3_‐GSH). Then, glutathionylation stimulated by treatment of the cells with glucose oxidase (ROS stimulus) was performed, followed by cell lysis and incubation of the lysate containing azide groups with biotin‐alkyne under specific conditions of the click reaction. The glutathionylated proteins were enriched on streptavidin‐agarose beads, the proteins bound to beads were on‐bead digested by trypsin/Lys‐C and the resulting glutathionylated peptides were eluted from beads and analyzed by LC–MS/MS. To cleave the biotin from glutathionylated peptides, and thus obtain smaller fragments of modification to the glutathione derivative conjugated to cysteine residue, cleavable biotin‐alkyne was used. Three biotin‐alkynes (biotin‐DADPS‐alkyne, biotin‐DDE‐alkyne and biotin‐PC‐alkyne) were tested, each containing a linker with different conditions for a cleavage (i.e. cleavage under acidic condition, cleavage using hydrazine with neutral pH and cleavage by application of irradiation at 365 nm, respectively) [33]. However, the DADPS linker performed best in terms of number of glutathionylated peptides identified by LC–MS/MS analysis. Figure 2 shows a schematic for the clickable glutathione approach applied in VanHecke *et al*. [33]. Using this approach, about 1000 glutathionylated proteins were identified with over 1700 glutathionylated peptides with specific cysteine sites. MS data were subjected to bioinformatics and cluster analysis and this resulted in the identification of 125 glutathionylated proteins, for which the impaired functions were connected to cardiomyopathy (e.g. sarcomeric contractile and structural proteins, chaperones and mitochondrial metabolism proteins). The study included implications about functional changes of identified sarcomere‐associated protein CSRP3 and three mitochondrial complexes (I, II and III) based on the identification of their glutathionylated cysteine sites and the available structural information [33].

A global profiling of reversible/irreversible cysteine redox PTMs of proteins in rat hearts during myocardial ischemia and I/R has been reported [50]. Herein, both the enrichment at peptide level and quantitative MS allowed to profile reversible redox PTMs in the presence/absence of *N*‐2‐mercaptopropionylglycine (MPG; aminothiol antioxidant) for antioxidant remedy of contractile dysfunction. In addition, parallel reaction monitoring‐MS enabled quantitative identification of irreversible PTMs (cysteine‐SO_2_H/SO_3_H). Over 1370 reversibly oxidized cysteine peptides were significantly regulated by ischemia and/or I/R. About 219 peptides were identified as irreversible modified by cysteine‐SO_2_H/cysteine‐SO_3_H (34 peptides relatively quantified by parallel reaction monitoring‐MS), predominantly, after I/R. Many functionally significant sites were found to be protected from both reversible and irreversible redox modifications by adding MPG during reperfusion. In summary, this approach allowed quantitation of both reversible and irreversible PTMs on protein redox sites during I/R and after antioxidant intervention. This study demonstrated that a variety of cysteine redox PTMs (targets of reactive oxygen species) jointly contributed to the pathogenesis of I/R [50].

A high‐throughput method has been applied to characterize cardiac palmitoylome (S‐palmitoylated proteins) and to identify palmitoylating DHHC enzymes expressed in human, dog and rat hearts [65]. Resin‐assisted enrichment of S‐palmitoylated proteins from membrane fractions of heart tissue samples followed by LC–MS/MS identification enabled the detection of representative cardiac palmitoylome consisting of 454 palmitoylated proteins (each protein had to be present at least in two species). Compared to human palmitoylomes of non‐cardiac cell types that were published in the SwissPalm database (2587 proteins) [48], 79% of 454 proteins identified in [65] overlapped. In case mouse non‐cardiac cell types from the SwissPalm database were considered as well, the overlap was 90%. In addition, 11 DHHC enzymes were detected and validated in the heart across these species by probing whole tissue lysates from human, dog and rat hearts [65].

**Fig. 3:**
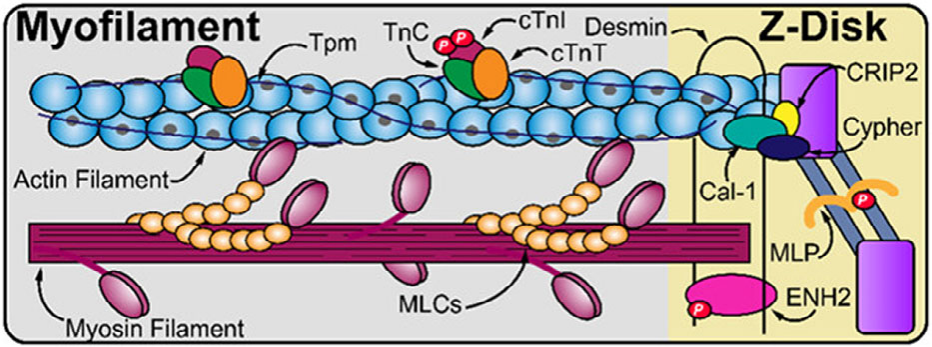
The structure of sarcomere, the basic contractile unit of the heart, showing the proteins and their altered phosphorylation that were identified in ischemic cardiomyopathy (ICM) patients. The sarcomere contains thin actin‐based filament and thick myosin‐based filament laterally bordered by the Z‐disk. Decreased expression and phosphorylation levels of cardiac troponin I (cTnI) and enigma homolog 2 (ENH2) were detected, as well as increased phosphorylation of muscle LIM protein (MLP) and calsarcin‐1 (Cal‐1) in ICM cardiac tissues. Other proteins depicted: cysteine‐rich protein 2 (CRIP2), cardiac troponin T (cTnT), tropomyosin (Tpm), myosin light chains (MLCs) and troponin C (TnC). Figure adapted from Chapman *et al*. [1]; copyright (2023) American Chemical Society.

MS‐based proteomics has been used for simultaneous identification/quantitation of sarcomeric proteoform changes in failing hearts of end‐stage ischemic cardiomyopathy (ICM) patients and healthy donors [1]. Herein, the alteration in phosphorylation and expression levels of several sarcomeric proteins was detected in extracted tissues between both groups; namely, the significant decrease in phosphorylation and expression levels of enigma homolog 2 (ENH2) and cardiac troponin I (cTnI) and the significant increase in phosphorylation of Z‐disk proteins calsarcin‐1 (Cal‐1) and LIM protein (MLP) in ICM patients. Furthermore, the data revealed significantly varied expression of isoforms of skeletal α‐actin (up‐regulation) and β‐tropomyosin (down‐regulation) in ICM tissue. In conclusion, this was the first study that clarified the alteration of sarcomeric subproteome in ICM patients using a MS‐proteomic approach and it highlighted dysregulation of sarcomere function on molecular level [1]. Figure 3 shows a schematic of the sarcomere with the proteins for which expression and phosphorylation levels were quantified in ICM patients [1].

The changes in cardiac protein lysine acetylation during heart enlargement and fibrosis by diet‐induced obesity have been investigated [84]. It is well known that accumulation of fat leads to remodeling of myocardium (i.e. increase in ventricular wall thickness and fibrosis and, eventually, in HF) [129]. In the study by Romanick *et al*. [84], a rodent model of obesity‐induced remodeling (C57BL/6J mice) was evaluated using TMT‐tagged MS (TMT 10‐plex isobaric label kit; Thermo Fisher Scientific, Waltham, MA, USA). Over 2500 proteins were detected in protein lysates from the left ventricle of the myocardium, of which 65 proteins were significantly altered by diet‐induced obesity (*p* < 0.1). For example, malonyl‐CoA decarboxylase and pyruvate dehydrogenase kinase isozyme 4 were up‐regulated. Ingenuity Pathway Analysis (Qiagen, Redwood City, CA, USA) demonstrated the association of these proteins with metabolic dysfunction, cardiovascular disease, regulation of fibrosis, cardiac enlargement and HF. In addition, 189 proteins were acetylated and acetylation levels of 14 proteins were significantly changed (*p* < 0.1) by obesity. Out of these 14 proteins, acetylation of nine proteins and five proteins was down‐regulated and up‐regulated, respectively; increased acetylation was found in both aconitase hydratase 2 and dihydrolipoyl dehydrogenase. By Ingenuity Pathway Analysis, 47 acetylated proteins out of 189 were part of the cardiovascular disease pathway. Acetylated proteins were mostly involved in muscle contractility and energy metabolism. For mitochondrial proteins, acetylation was mainly up‐regulated, whereas, for sarcomeric proteins, acetylation was down‐regulated. In summary, this study implied the essential role of cardiac acetylation in obesity‐mediated remodeling and it contributed to the knowledge about a potential role of non‐histone acetylation in this cardiac disorder [84].

A recent study has reported on citrullination (deimination) of the proteins in multiple organs, including the heart, detected by MS‐based proteomics [130]. Herein, the mouse hyper‐citrullinated spectral library was developed for confident identification and validation of citrullinated protein/peptide sites. In the heart, the lowest amount of citrullinated sites were detected (about 800) with highest number of citrullinated residues in the brain (about 1400) and moderate number of citrullinated sites in kidney, lung and liver (around 980, 1180 and 1180, respectively). After subjecting all citrullinated proteins to Gene Ontology (http://geneontology.org) analysis, around 41% of them exhibited an association with catalytic activities, 38% with binding, 9% with structural molecular activity, 6% with transporter activity and 5% with molecular function regulation. In summary, over 90% of citrullinated proteins and sites have not been reported previously, and a four‐fold increase in citrullinome coverage was reached compared to currently applied techniques. Most citrullinated proteins were tissue‐specific and citrullination showed specialized signaling networks that are yet not known [130].

Proteome map of PTMs has been performed in human hearts using MS measurements and PTM identification algorithms [131]. Herein, more than 150 different PTMs in three chambers of human myocardium (right atria, left atria and left ventricle) were identified using Comet‐PTM search engine [101]. In the study by Bagwan *et al*. [131], the PTMs of selected proteins, for which dysfunction was associated with cardiac pathologies, were discussed in terms of well‐known modifications (acetylation and phosphorylation), as well as less tested modifications (methylation, oxidation and nitrosylation). This included myosin heavy chains α and β (MYH6 and MYH7), troponin I (TNNI3), ryanodine receptor 2 (RYR2) and phospholamban (PLN). For example, in MYH6, oxidation and methylation were frequent PTMs; however, novel phosphorylation sites (S1544, S1650 and S1734), acetylation sites (K1559, K1653 and K1816) and glycosylation sites (K1018 and K1571) were identified on MYH6. The study provided a global map of PTMs present in human heart with PTMs and modified amino acid sites that were not yet studied in relation to cardiac proteins.

### Proteomics targeting specific proteins

Methods for the analysis of intact cardiac troponins and their PTMs have been published recently and include top‐down MS‐based proteomic approach [2]. Regulatory proteins cTnI and cardiac troponin T (cTnT) are components of the cTn that are used as clinical biomarkers of cardiac injury [132]. Their various PTMs (e.g. phosphorylation) [133, 134] altered protein–protein interactions within the sarcomere, they modulated heart contractility and they were involved in function alteration during cardiac pathologies. In a study by Tiambeng *et al*. [2], two protocols were described for preparation of cardiac troponin PTM proteoforms from cardiac tissue (i.e. myofilament‐enriched protein extraction and immunoaffinity purification). Top‐down on‐line LC/MS was used for separation, profiling and quantitation of troponin proteoforms and FT‐ICR MS was applied for characterization of troponin sequence and the location of PTMs. Freely‐available mash software developed was used for proteoform identification and PTM characterization [2].

The goals of the next study were quantitation of the non‐enzymatic glycation levels of human serum albumin in the plasma of the non‐diabetic patients with HF and testing biological effects of glycated‐human serum albumin (GA) on HL‐1 cardiomyocytes *in vitro* [135]. Using MS‐based proteomics, it was confirmed that GA levels increased significantly in plasma of HF patients compared to healthy controls, as well as with increasing severity of HF. Moreover, the treatment of HL‐1 cells by GA *in vitro* (16 h with 250 μg·mL^−1^ GA) caused the secretion of the proteins that are known to be associated with the response to stress, and with altered levels compared to control cells (e.g. nucleolin and heat shock protein HSP90 beta were up‐regulated; fold change > 1.5). Furthermore, GA treated HL‐1 cardiomyocytes showed an increase in ROS formation and enhanced levels of pro‐inflammatory and pro‐oxidant proteins (e.g. cytokine interleukin‐6 and tumor necrosis factor α). This study highlighted the importance of MS in the identification/quantitation of PTM such as glycation, and confirmed the pro‐oxidant and pro‐inflammatory effects of GA on cardiomyocytes in the pathogenesis of HF [135].

**Fig. 4:**
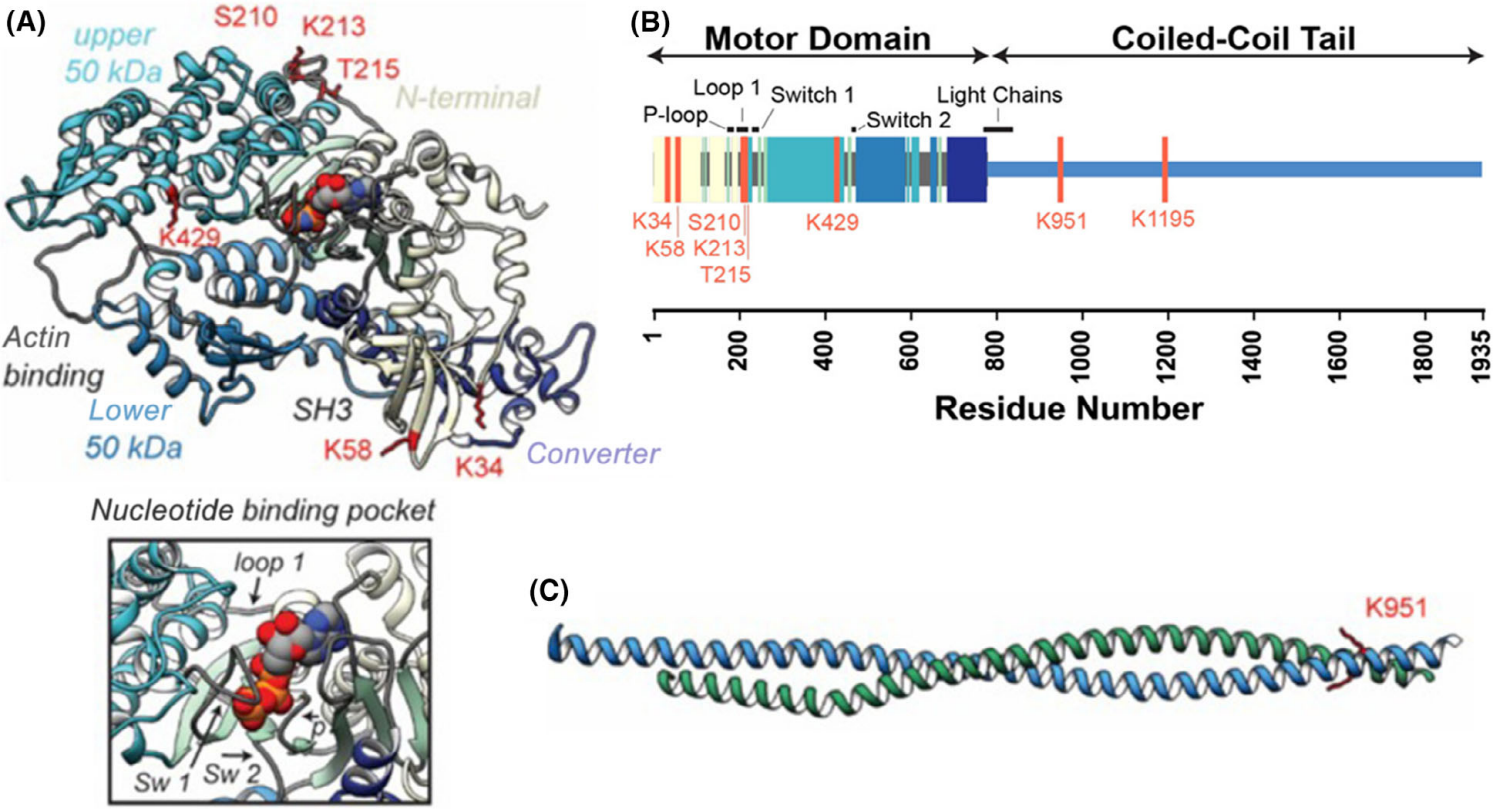
The structural model and sequence of β‐myosin heavy chain (β‐MHC) with newly identified post‐translational modification (PTM) sites. (A) X‐ray crystal structure of myosin motor domain; the four motor subdomains [N‐terminal domain (yellow), upper 50 kDa domain (cyan), lower 50 kDa domain (light blue) and converter domain (dark blue)] are shown with identified PTMs (acetylation and phosphorylation) in red colors (K213‐Ac, K429‐Ac, K58‐Ac, K34‐Ac, S210‐P and T215‐P); the inset highlights the nucleotide binding pocket and functional loops (loop 1, switches 1 and 2, and phosphate binding loop). (B) Schema of β‐MHC sequence with key functional regions and locations of PTMs; colored blocks correspond to motor subdomains shown in (A). (C) X‐ray crystal structure of S2 region of myosin coiled‐coil tail with K951‐Ac site depicted (blue, chain A; green, chain B). Figure adapted from Landim‐Vieira *et al*. [136].

Furthermore, the potential role of novel PTM sites in functional regions of sarcomeric β‐myosin heavy chain (β‐MHC) on cardiac muscle performance has been investigated [136].The bottom‐up MS‐based proteomics (SDS/PAGE, in‐gel digest and LC–MS/MS) has been used to identify and quantify acetylation and phosphorylation of β‐MHC in human healthy donor hearts (non‐failing; NF) and end‐stage heart failure patients [ischemic HF (I‐HF) or non‐ischemic HF (NI‐HF)] [136]. Altogether, six acetylated lysines (K34‐Ac, K58‐Ac, K213‐Ac, K429‐Ac, K951‐Ac and K1195‐Ac) and two phosphorylated sites (serine S210‐P and threonine T215‐P) were identified/quantified. The occurrence of these novel PTM sites on β‐MHC in NF hearts implied that they were essential for normal function of the protein and muscle filaments containing it [136]. These PTM sites showed a tendency for downregulation in failing hearts compared to NF hearts. For example, K951‐Ac was significantly down‐regulated in both I‐HF and NI‐HF compared to NF samples. In addition, molecular dynamics simulations were carried out and focused on four PTMs to clarify their functions in β‐MHC; namely, K951‐Ac within the S2 region, K58‐Ac within the SH3‐like domain and K213‐Ac/T215‐P (doubly modified peptide) close to the ATP‐binding pocket. Figure 4 shows β‐MHC structural model (A, motor domain; C, S2 region of myosin tail) with newly identified PTMs. From the results gathered by molecular dynamics simulation, it can be concluded that PTMs can potentially modulate dynamics in β‐MHC; however, whether their downregulation in I‐HF and NI‐HF hearts means a loss of beneficial regulation of myofilament function remain to be confirmed [136].

Table 1 summarizes the types of PTMs covered in this section that have been identified in the heart from 2018 onward and are associated with cardiovascular disorders/diseases using MS‐based proteomic approaches. A short description of the findings is also included.

**Table 1:** The examples of post‐translational modifications (PTMs) that have been identified recently in the heart (2018–2023) and associated with cardiovascular disorders/diseases using MS‐based proteomic approaches.

Type of PTM	Cardiovascular disorder/disease	Findings	Reference
Crotonylation	Cardiac hypertrophy and arrhythmia	Correlation was confirmed between impaired cardiac function, hypertrophy and arrhythmia and increased SERCA2a crotonylation mediated by SIRT1 in mice model	[52]
Succinylation	Cardiac hypertrophy	Phenotype of increased mortality in whole‐body SIRT5 KO mice model subjected to pressure overload‐induced hypertrophy by TAC surgery was not repeated in cardiomyocyte‐specific SIRT5 KO mouse model; ablation of SIRT5 resulted in accumulation of protein succinylation, however, no difference in survival between cardiomyocyte‐specific SIRT5 KO mice after TAC surgery and their littermate controls was found	[116]
Succinylation	AF	The alteration in energy metabolism‐associated protein levels and succinylation levels between AF patients and patients without AF implied that improving myocardial energy metabolism can be a way to treat AF	[118]
Succinylation	AAD	Succinylation contributed to aortic diseases and succinylated proteins can function as potential markers and therapeutic targets for patients with thoracic aortic aneurysm and thoracic aortic dissection	[91]
Malonylation	Cardiac hypertrophy	Protein malonylation levels were reduced in both TAC surgery‐induced cardiac hypertrophy mice heart and H9C2 hypertrophic cardiomyocytes *in vitro* implying association of cardiac hypertrophy with downregulation of protein malonylation	[32]
Glutathionylation	Cardiomyopathy	In HL‐1 cells, 125 glutathionylated proteins were identified, whose impaired functions were related to cardiomyopathy	[33]
Redox PTMs	Ischemia and I/R injury	Quantitation of both reversible and irreversible (cysteine‐SO_2_H/SO_3_H) PTMs on protein redox sites during I/R was performed; the presence/absence of aminothiol antioxidant MPG was used for antioxidant remedy of heart contractile dysfunction; variety of cysteine redox PTMs jointly contributed to I/R in rat hearts	[50]
S‐palmitoylation	No disease	Representative cardiac palmitoylome (454 palmitoylated proteins) was established from the human, dog and rat hearts; 11 palmitoylating DHHC enzymes were detected and validated in the hearts across these species	[65]
Phosphorylation	ICM	Alterations in sarcomeric subproteomes of ICM patients pointed to dysregulation of the sarcomere function; significant decreases in both phosphorylation and expression levels of ENH2 and cTnI were identified, as well as significant increases in phosphorylation of Z‐disk proteins Cal‐1 and MLP in ICM patients	[1]
Acetylation	Heart enlargement and fibrosis	Acetylation levels of nine proteins were significantly down‐regulated and five proteins significantly up‐regulated in mice model of obesity‐induced remodeling; essential role of non‐histone acetylation in heart enlargement and fibrosis was confirmed	[84]
Citrullination	No disease	The mouse hyper‐citrullinated spectral library was developed for identification of citrullinated proteins in multiple organs in MS‐based proteomics; the number of citrullinated sites decreased in order brain > liver, lung > kidney > heart	[130]
Glycation	HF	Glycated‐human serum albumin (GA) levels increased significantly in plasma of HF patients compared to healthy control and with increasing severity of HF; pro‐oxidant and pro‐inflammatory effects of GA on HL‐1 cells were detected in pathogenesis of HF	[135]
Acetylation and phosphorylation	Ischemic HF and non‐ischemic HF	Six acetylated lysine sites and two phosphorylated sites were identified and quantified on β‐MHC in both healthy donors (NF) and HF patients; these PTMs exhibited a tendency for downregulation in HF patients compared to NF hearts	[136]

## Concluding remarks and future directions

Advances in experimental techniques and instrumentation supported by biostatistical and computational strategies in proteomics have enabled the characterization of PTM profiles in various pathologies. In recent years, increasing number of MS‐based proteomic studies has been dedicated to cardiovascular disease‐specific PTMs and the determination of PTM types and their localizations within a protein amino sequence. In addition, emerging roles of higher‐level regulation by PTMs in PTM‐associated protein–protein interaction and PTM crosstalk in the heart were explored [137]. All of these efforts have already contributed to clarification of the mechanisms in which specific PTMs dynamically regulated physiological and pathological situations at the molecular level. Ultimately, these results can lead to further recognition of potential clinical markers of heart disorders and the development of effective treatments in the future.

However, the experiments performed in laboratories which simulate and model biological/pathological conditions do not necessarily reflect the true situation occuring during varied circumstances. For example, the high concentration of glucose oxidase that has been used to identify high number of glutathionylated proteins *in vitro* revealed the proteins that may be susceptible to glutathionylation only at high levels of oxidative stress; however, these high levels of H_2_O_2_ produced by glucose oxidase may not be achievable in a biological environment [33]. In addition, during sample processing in MS‐based proteomics, chemical modification can be added [100], PTMs can be altered and /or unstable PTMs can be lost [138], and thus incorrect conclusions can be made from the experimental data. In the biopsy samples from patients, factors such as age, additional disorders, subjection to drugs and presence in various environments can misrepresent the results. Computational methods can point out and predict all possible sites in the protein amino acid sequence that can be modified, and this is an important step in PTM identification; however, they should be critically evaluated for their weaknesses, and the PTMs that are relevant to particular pathological condition tested and validated in a real biological setting. Thus, all of these issues need to be taken into consideration and addressed. For example, proteomic data should be confirmed by biochemical and/or physiological methods to validate them and to gather complementary information that can create a more complete picture. The major challenges that remain are confirmation of the experimental data in clinical trials with large patient cohorts and transfer of the knowledge about the therapeutic relevance of PTMs in various stages of cardiac disorders to the clinic.

## Conflicts of interest

The author declares that he has no conflicts of interest.

## Data Availability

The data generated during the current study are available from the corresponding author upon reasonable request.
